# Supplementary material for: Age-related differences in human cortical microstructure depend on the distance to the nearest vein
Source: Brain Commun. 2024 Sep 19;6(5):fcae321. doi: 10.1093/braincomms/fcae321 (PMC11443451; doi:10.1093/braincomms/fcae321)
Supplement: fcae321_Supplementary_Data [file fcae321_supplementary_data.pdf]

Supplementary Material

Supplementary Methods

Tactile detection task

Tactile detection thresholds of the right index fingertip were assessed using fine hair stimuli (Semmes-Weinstein monofilaments; Baseline, Fabrication Enterprises Inc., White Plains, NY, USA; stimulus intensities: 0.008 g, 0.02 g, 0.04 g, 0.07 g, 0.16 g, 0.4 g, 0.6 g, 1.0 g, 1.4 g, 2.0 g). Stimuli were manually applied to a predefined skin area (circle with 2 mm diameter) at an angle of approximately 90 degree, for one second.[1] Stimulus application was guided by auditory cues (Psychophysics Toolbox for MATLAB R2017b).[2] All participants sat in front of a screen that signalled the beginning and ending of stimulus intervals and listened to white noise via headphones. The right hand (palm facing upwards) was fixated on a pillow behind a paper wall, preventing participants from seeing their own hand and the experimenter. In a two-alternative forced choice paradigm, participants chose one of two possible time intervals that contained the stimulation (randomly applied in the first or second interval).[3,4] Application of tactile monofilaments followed a 3-down/1-up staircase with two interleaved staircases, one starting at a 0.02 gram and the other starting at 0.4 gram. The stimulus weight was increased by one step after each error, and decreased by one step after every three correct responses, until stable performance was reached.[4] The experiment was finished when for the last 30 trials the standard variation in stimulus intensity was 1 step or less[4], or when the maximum number of 100 trials was reached. The participant‘s tactile detection threshold was defined as the mean stimulus intensity across reversal points (change of response from correct to incorrect or incorrect to correct) within the period of stable performance (i.e., the last 10 trials).[3] The experiment took approximately 12 minutes. Stimulus intensities were transformed logarithmically on a 1/10th milligram scale (log 10 0.1mg). Lower values indicate higher tactile sensitivity to mechanical forces.

Two-point discrimination task

We also assessed tactile 2-point discrimination (2PD) performance of the right index fingertip.[5–7] Stimulation was applied by two rounded pins (diameter=0.4 mm) simultaneously touching the skin. A custom-made, fully automatic stimulation device moved the pins up and down (controlled by Presentation version 16.5, Neurobehavioral Systems, Inc., Albany, CA, USA). The amplitude of pin movement was adjusted to the individual detection threshold, but

was at least set to 1.2 mm. Spacing between pins ranged from 0.7 to 2.8 mm (in steps of 0.3 mm) for younger adults and from 0.7 to 6.3 mm (in steps of 0.8 mm) for older adults. A single pin was included as control condition. Pin spacing was vertically adjusted by a rotating disc containing all possible conditions (n=9). In a two-alternative forced-choice paradigm, pin conditions were pseudo-randomly presented. Participants indicated whether they perceived one or two single pins touching their fingertip. They were instructed to give the answer “two pins felt” only if they were certain. The hand was covered by a white box during the task. Each run included 90 trials (10 repetitions per pin condition). Unique sequences of pin spacing conditions were used per participant and run. All participants completed two runs. Intertrial intervals were pseudo-randomized and varied between 1 to 5 seconds. 2PD thresholds were calculated based on the second run. Answers “two pins felt” were fitted as percentages across ascending pin distances. A binary logistic regression was used to fit the data (glmfit function, Statistics Toolbox for MATLAB R2017b). The 2PD threshold was taken from the pin distance where the 50 percent level crossed the fitted sigmoid curve.[5,6,8,9] Lower values indicate higher spatial acuity.

Sensorimotor integration

Sensorimotor integration was assessed with a custom made pressure sensor (held between the thumb and index finger of the right hand).[10] Reference forces that were to be matched ranged from 5% to 25% of the individual maximum grip force to avoid muscle fatigue.[11] Participants solved a visuo-motor matching task, demanding them to continuously adjust the grip force.[10,12] Applied forces were sampled at a frequency of 100 Hz and projected on screen at a refresh rate of 60 Hz. The task was controlled by the software package Presentation (version 16.5, Neurobehavioral Systems, Inc., Albany, CA, USA). Each trial contained a unique pseudorandomized sequence of 10 position changes at five different amplitudes (5%, 10%, 15%, 20%, 25% of maximum grip force), leading to a mean frequency of 0.25 Hz. After a period of task familiarisation[11,12], all participants performed the task for a total duration of 20 seconds. One run contained 15 trials with intertrial intervals of 10 seconds, lasting about 8 minutes in total. All participants performed two runs that were separated by a 5-minute resting period. After each trial, participants received feedback about their individual performance level on screen. We monitored the time (in seconds) the controllable bar was within a given percentage above (2.5%) and below (2.5%) the target line (upper edge of the reference bar)[10,11]. Higher values reflect better sensorimotor integration.

Supplementary Figures

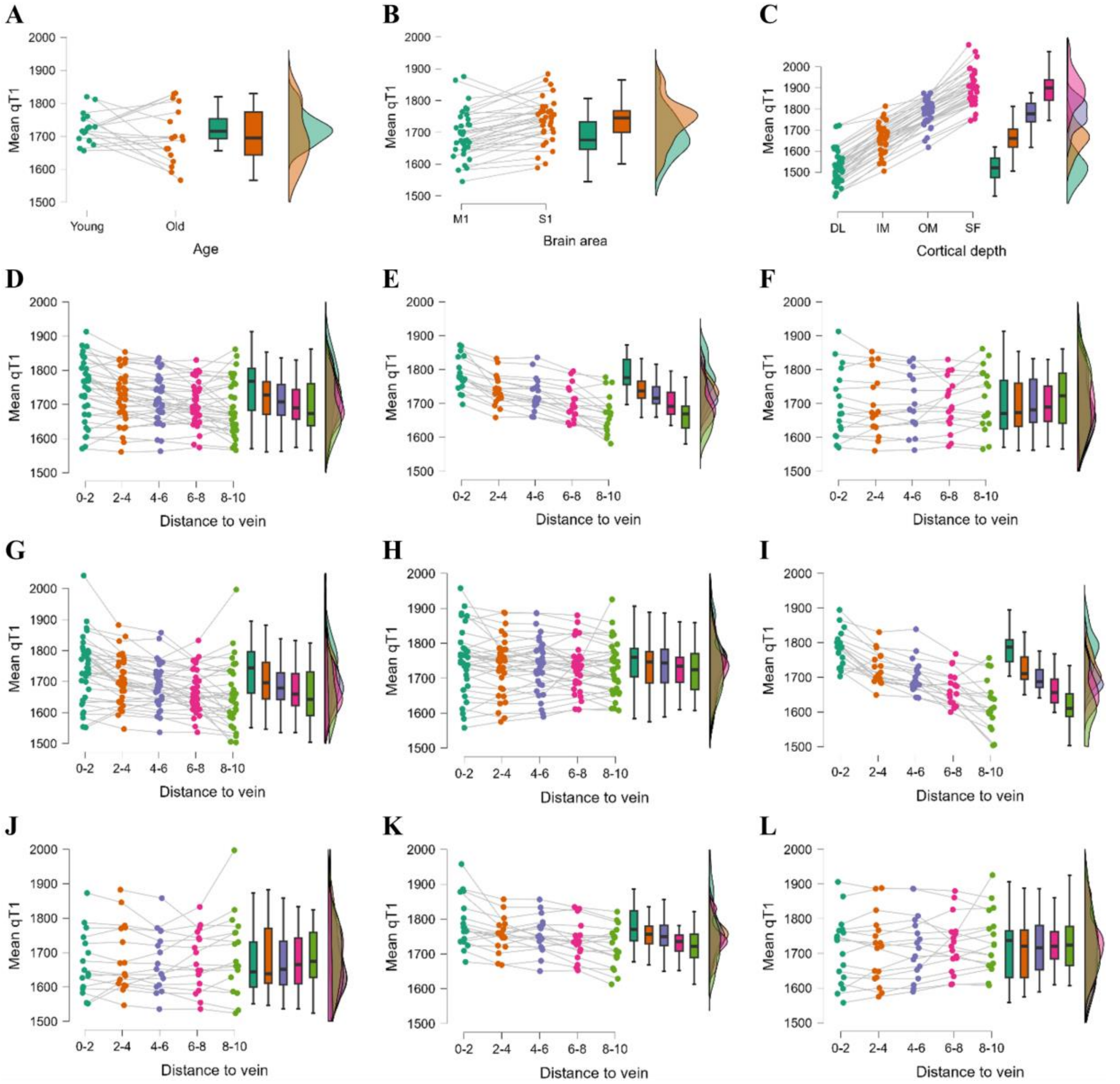

Supplementary Figure 1. Descriptive raincloud plots of individual quantitative T1 (qT1) values for tested ANOVA conditions. A: Younger versus older adults. **B:** M1 versus S1. **C:** Cortical depths. **D:** Distance to the nearest vein. **E:** Distance to the nearest vein for younger adults. **F:** Distance to the nearest vein for older adults. **G:** Distance to the nearest vein for M1. **H:** Distance to the nearest vein for S1. **I:** Distance to the nearest vein for younger adults in M1. **J:** Distance to the nearest vein for older adults in M1. **K:** Distance to the nearest vein for younger adults in S1. **L:** Distance to the nearest vein for older adults in S1. DL = deep cortical depth, IM = inner middle cortical depth, OM = outer middle cortical depth, SF = superficial cortical depth. M1 = primary motor cortex, S1 = primary somatosensory cortex. qT1 values given in milliseconds. Distance to the nearest vein given in millimetres.

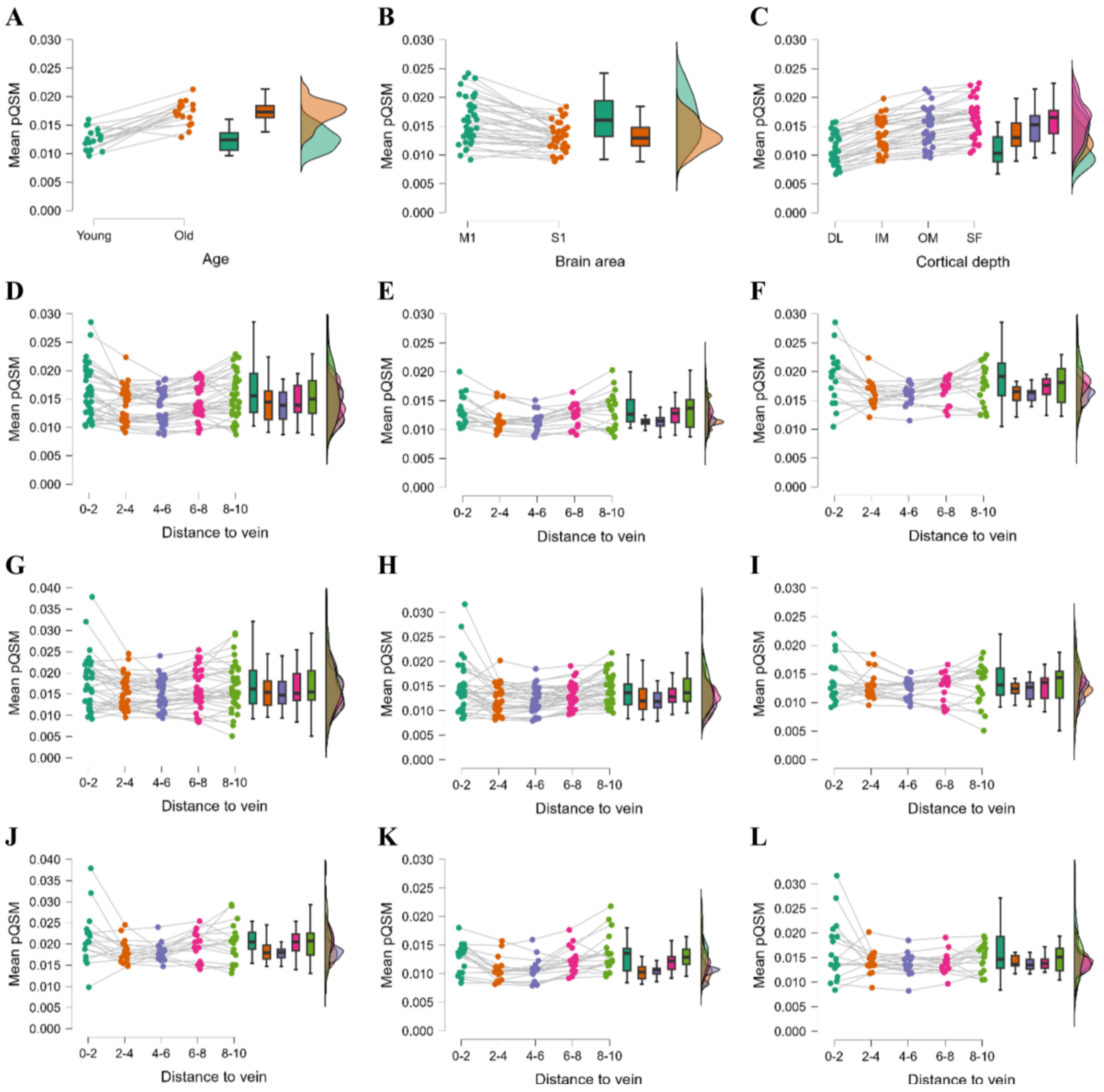

Supplementary Figure 2. Descriptive raincloud plots of individual positive QSM (pQSM) values for tested ANOVA conditions. A: Younger versus older adults. **B:** M1 versus S1. **C:** Cortical depths. **D:** Distance to the nearest vein. **E:** Distance to the nearest vein for younger adults. **F:** Distance to the nearest vein for older adults. **G:** Distance to the nearest vein for M1. **H:** Distance to the nearest vein for S1. **I:** Distance to the nearest vein for younger adults in M1. **J:** Distance to the nearest vein for older adults in M1. **K:** Distance to the nearest vein for younger adults in S1. **L:** Distance to the nearest vein for older adults in S1. DL = deep cortical depth, IM = inner middle cortical depth, OM = outer middle cortical depth, SF = superficial cortical depth. M1 = primary motor cortex, S1 = primary somatosensory cortex. pQSM values given in parts per million. Distance to the nearest vein given in millimetres.

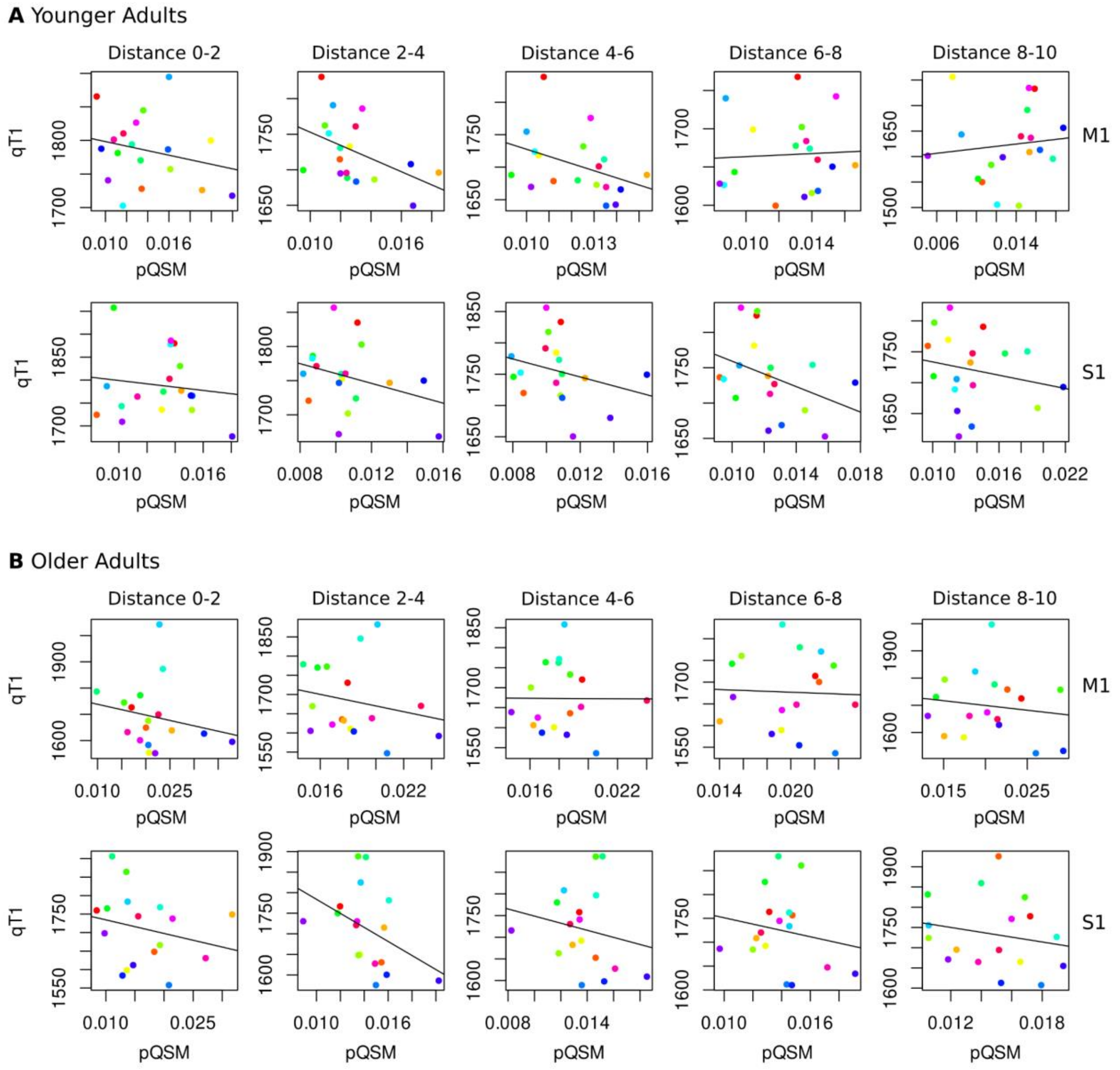

Supplementary Figure 3. Relationship between quantitative T1 (qT1) and positive QSM (pQSM) values. Descriptive scatterplots showing individual qT1 values (milliseconds, y-axis) and pQSM values (parts per million, x-axis) for each brain area (M1, S1) by distance condition (0-2 mm, 2-4 mm, 4-6 mm, 6-8 mm, 8-10 mm). Different colours indicate different participants. Regression lines were generated based on simple linear regression models (qT1 ~ pQSM). For exact correlation coefficients see **Supplementary Table 8**. **A:** Scatterplots for younger adults (n=18). **B:** Scatterplots for older adults (n=17). M1 = primary motor cortex, S1 = primary somatosensory cortex.

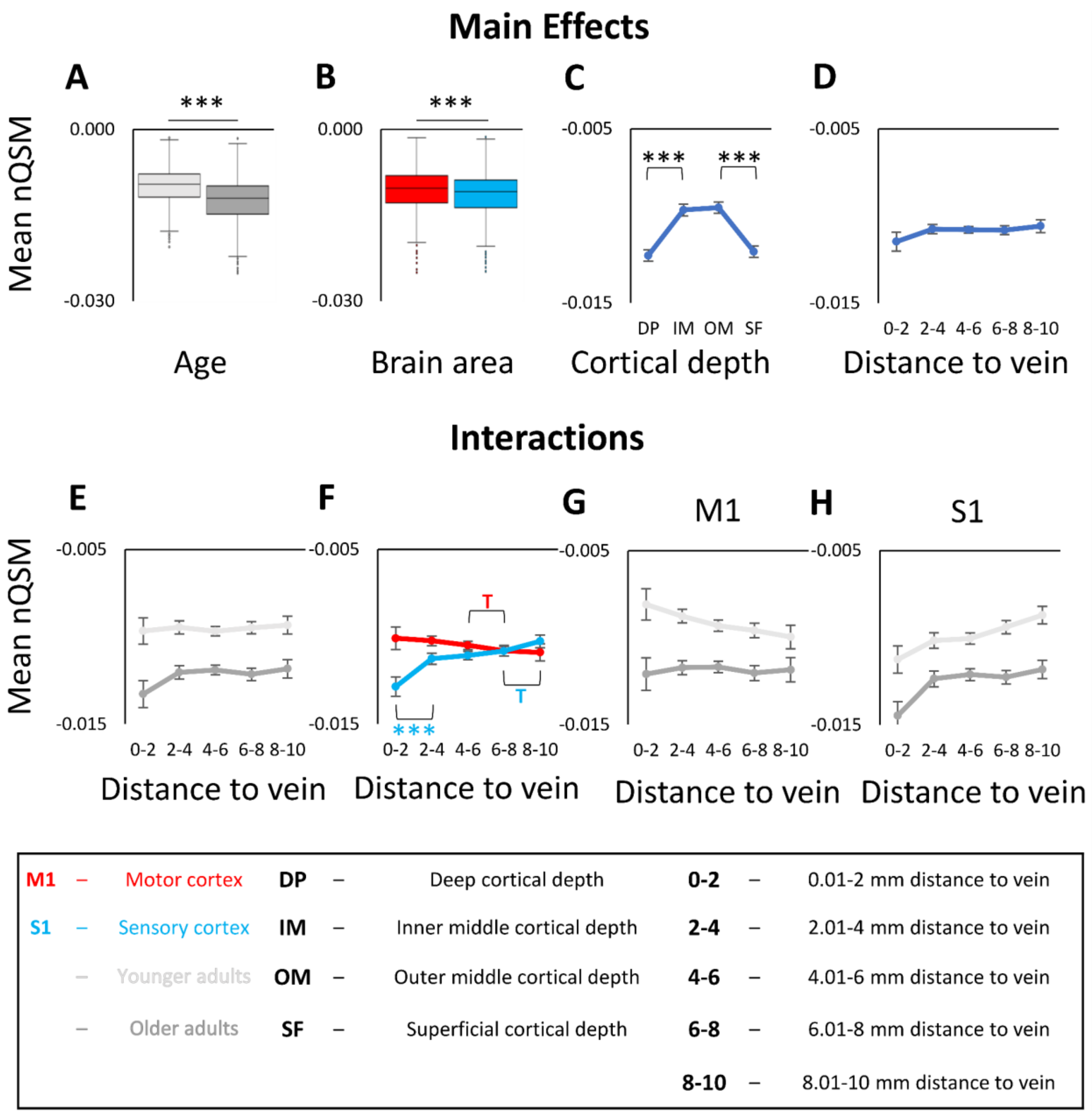

Supplementary Figure 4. ANOVA results for negative QSM (nQSM) values. A: Significant main effect of age on nQSM values. Shown are medians, interquartile ranges, and lower and upper quartiles for younger (n=18, *light grey*) and older (n=17, *dark grey*) adults. Dots mark outliers. nQSM values are given in parts per million (lower values indicate higher diamagnetic contrast). **B:** Significant main effect of brain area (primary motor cortex, M1 (*red*); primary somatosensory cortex, S1 (*blue*)) on nQSM values. **C:** Significant main effect of cortical depth on nQSM values. Dots indicate mean nQSM values for different cortical depths averaged across age groups, distances, and brain areas. Error bars indicate standard errors of the mean (SEM). **D:** No significant main effect of venous distance on nQSM values (0-2 = 0.01-2 mm, 2-4 = 2.01-4 mm, 4-6 = 4.01-6 mm, 6-8 = 6.01-8 mm, 8-10 = 8.01-10 mm). **E:** No significant interaction effect between venous distance and age on nQSM values. **F:** Significant interaction effect between venous distance and brain area on nQSM values. **G/H:** No significant interaction effect between venous distance, age and brain area (**G:** data for M1, **H:** data for S1). Significant results of post-hoc t-tests to follow-up mixed-effects ANOVA results (see **Supplementary Table 10** for statistical results) are marked by asterisks: * $p \leq 0.05$, ** $p \leq 0.005$, *** $p \leq 0.0005$ (uncorrected). Trends above

p=0.05 are marked by a T.

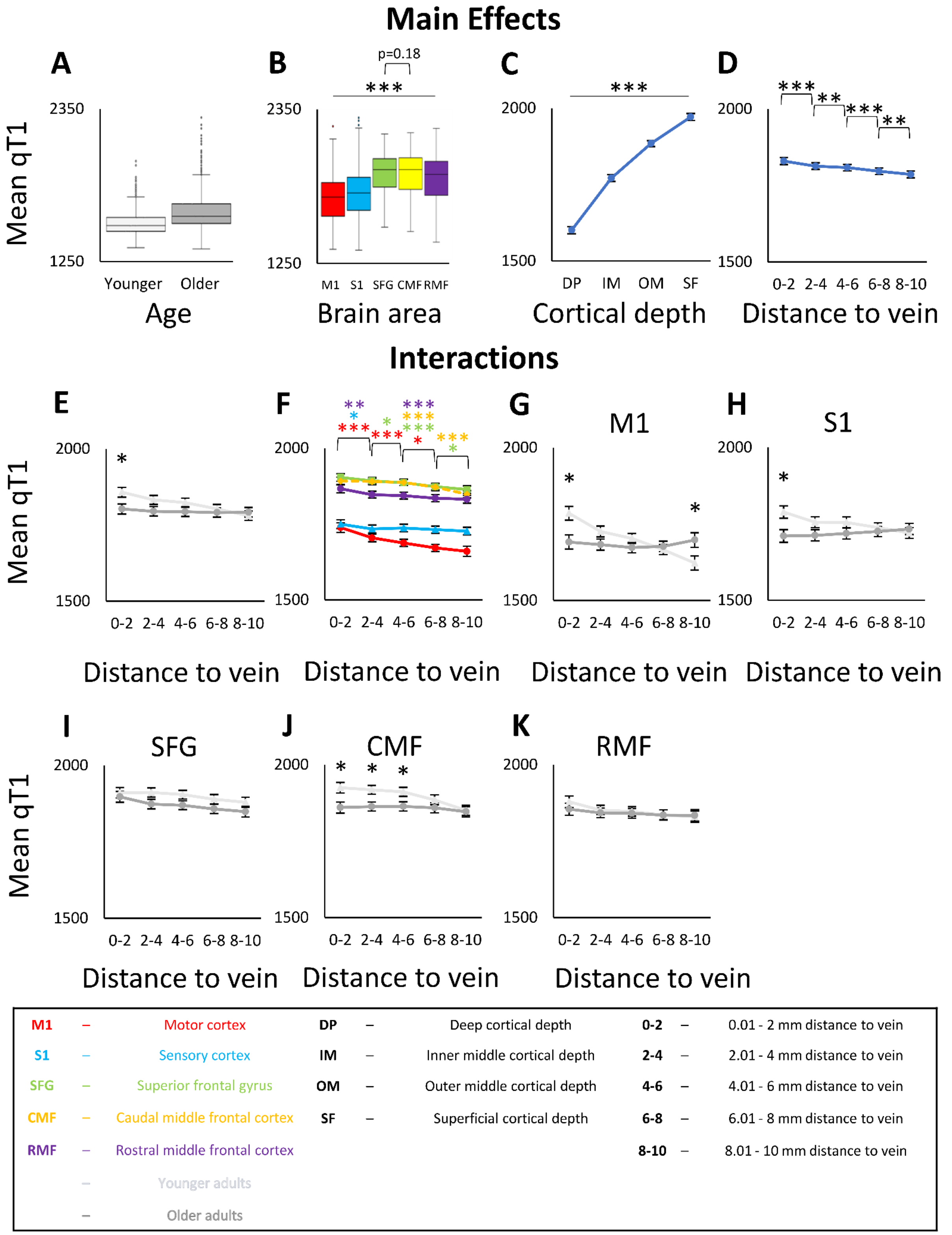

Supplementary Figure 5. ANOVA results for quantitative T1 (qT1) values including primary motor cortex (M1), primary somatosensory cortex (S1), superior frontal gyrus (SFG), caudal middle frontal cortex

(CMF) and rostral middle frontal cortex (RMF). qT1 values are given in milliseconds (lower values indicate higher myelin content). **A:** No significant main effect of age on qT1 values. Shown are medians, interquartile ranges, and lower and upper quartiles for younger (n=18, *light grey*) and older (n=17, *dark grey*) adults. Dots above a box mark outliers. **B:** Significant main effect of brain area (i.e., M1 (*red*), S1 (*blue*), SFG (*green*), CMF (*yellow*), RMF (*violet*)) on qT1 values. **C:** Significant main effect of cortical depth on qT1 values; dots represent mean qT1 values for the different cortical depths averaged across age groups, distances, and brain areas. Error bars show standard errors of the mean (SEM). **D:** Significant main effect of venous distance on qT1 values (0-2 = 0.01-2 mm, 2-4 = 2.01-4 mm, 4-6 = 4.01-6 mm, 6-8 = 6.01-8 mm, 8-10 = 8.01-10 mm). **E:** Significant interaction effect between venous distance and age on qT1 values. **F:** Significant interaction effect between venous distance and brain area on qT1 values. **G-K:** Significant interaction effect between venous distance, age and brain area (**G**: data for M1, **H**: data for S1, **I**: data for SFG, **J**: data for CMF, **K**: data for RMF). Significant results of post-hoc t-tests to follow-up mixed-effects ANOVA results (see **Supplementary Table 12** for statistical results) are marked by asterisks: * $p \leq 0.05$, ** $p \leq 0.005$, *** $p \leq 0.0005$ (uncorrected). Trends above $p=0.05$ are marked by a T.

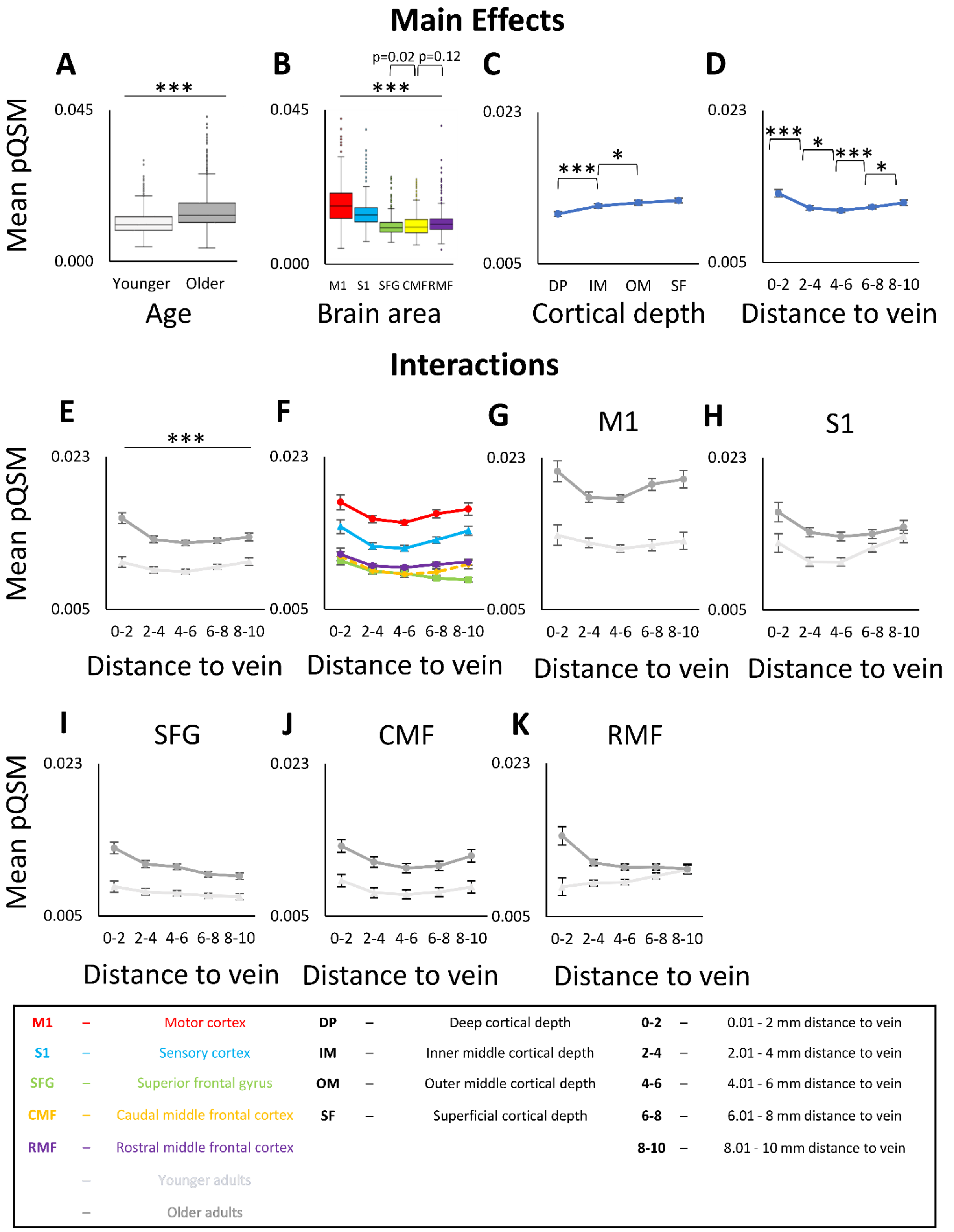

Supplementary Figure 6. ANOVA results for positive QSM (pQSM) values including primary motor cortex (M1), primary somatosensory cortex (S1), superior frontal gyrus (SFG), caudal middle frontal cortex (CMF) and rostral middle frontal cortex (RMF). A: Significant main effect of age on pQSM values. Shown are medians, interquartile ranges, and lower and upper quartiles for younger (n=18, *light grey*) and older (n=17, *dark grey*) adults. Dots mark outliers. pQSM values are given in parts per million (higher values indicate higher iron content). **B:** Significant main effect of brain area (M1 (*red*), S1 (*blue*), SFG (*green*), CMF (*yellow*),

RMF (*violet*)) on pQSM values. No significant effect for SFG-CMF and CMF-RMF comparisons. **C:** Significant main effect of cortical depth on pQSM values. Dots indicate mean pQSM values for different cortical depths averaged across age groups, distances, and brain areas. Error bars indicate standard errors of the mean (SEM). **D:** Significant main effect of venous distance on pQSM values (0-2 = 0.01- 2 mm, 2-4 = 2.01-4 mm, 4-6 = 4.01-6 mm, 6-8 = 6.01-8 mm, 8-10 = 8.01-10 mm). **E:** Significant interaction effect between venous distance and age on pQSM values. **F:** No Significant interaction effect between venous distance and brain area on pQSM values. **G-K:** No significant interaction effect between venous distance, age and brain area (**G:** data for M1, **H:** data for S1, **I**: data for SFG, **J**: data for CMF, **K**: data for RMF). Significant results of post-hoc t-tests to follow-up mixed-effects ANOVA results (see **Supplementary Table 13** for statistical results) are marked by asterisks: * $p \leq 0.05$, ** $p \leq 0.005$, *** $p \leq 0.0005$ (uncorrected). Trends above $p=0.05$ are marked by a T.

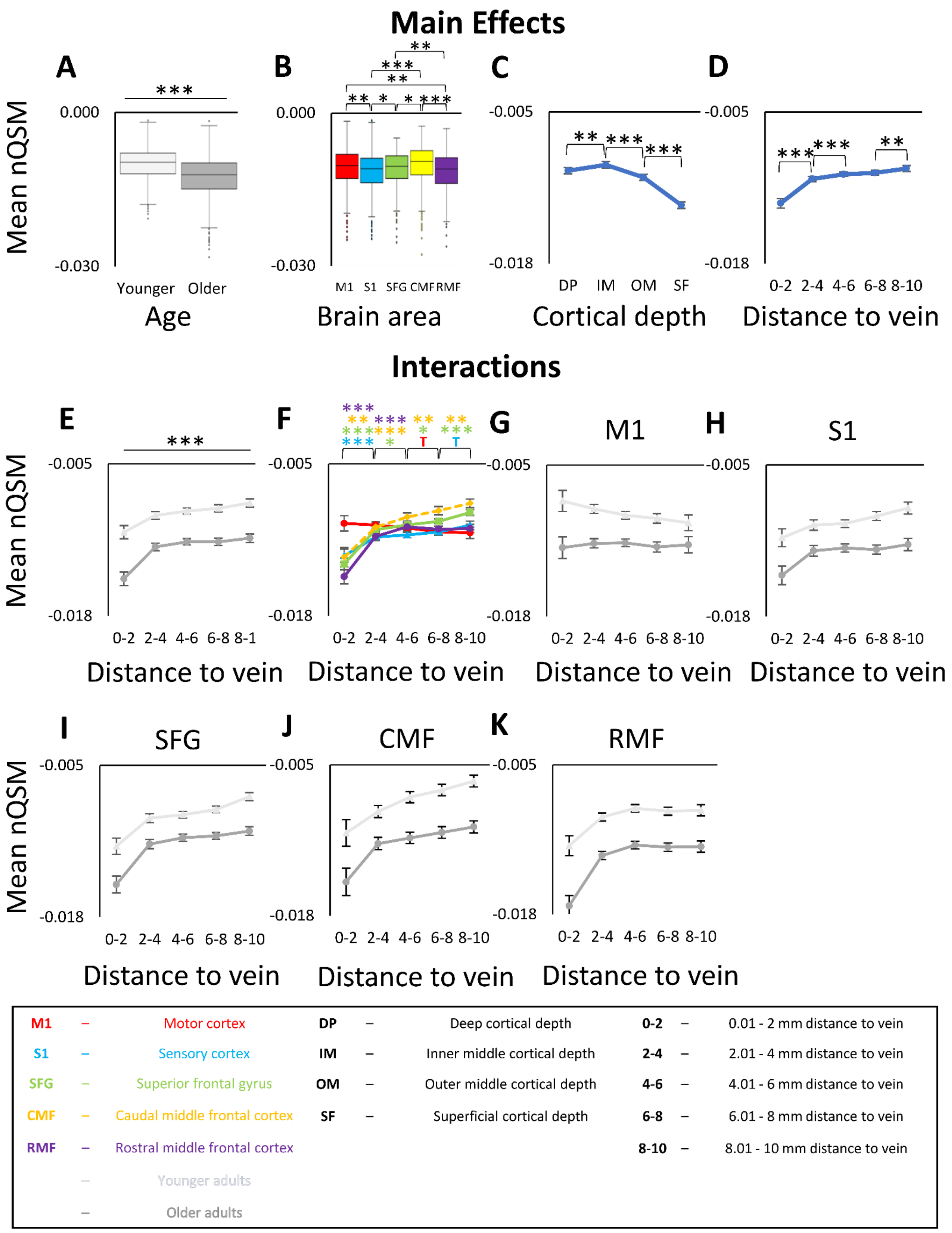

Supplementary Figure 7. ANOVA results for negative QSM (nQSM) values including primary motor cortex (M1), primary somatosensory cortex (S1), superior frontal gyrus (SFG), caudal middle frontal cortex (CMF), rostral middle frontal cortex (RMF). A: Significant main effect of age on nQSM values. Shown are medians, interquartile ranges, and lower and upper quartiles for younger (n=18, *light grey*) and older (n=17, *dark grey*) adults. Dots mark outliers. nQSM values are given in parts per million (lower values indicate higher diamagnetic contrast). **B:** Significant main effect of brain area (M1 (*red*), S1 (*blue*), SFG (*green*), CMF

(*yellow*), RMF (*violet*)) on nQSM values. **C:** Significant main effect of cortical depth on nQSM values. Dots indicate mean nQSM values for different cortical depths averaged across age groups, distances, and brain areas. Error bars indicate standard errors of the mean (SEM). **D:** Significant main effect of venous distance on nQSM values (0-2 = 0.01- 2 mm, 2-4 = 2.01-4 mm, 4-6 = 4.01-6 mm, 6-8 = 6.01-8 mm, 8-10 = 8.01-10 mm). **E:** Significant interaction effect between venous distance and age on nQSM values. **F:** Significant interaction effect between venous distance and brain area on nQSM values. **G-K:** No significant interaction effect between venous distance, age and brain area (**G**: data for M1, **H**: data for S1, **I**: data for SFG, **J**: data for CMF, **K**: data for RMF). Significant results of post-hoc t-tests to follow-up mixed-effects ANOVA results (see **Supplementary Table 14** for statistical results) are marked by asterisks: * $p \leq 0.05$, ** $p \leq 0.005$, *** $p \leq 0.0005$ (uncorrected). Trends above $p=0.05$ are marked by a T.

Supplementary Tables

Task	Younger Adults			Older Adults			
	Mean ± SD	Value Range	# Extreme Outliers	Mean ± SD	Value Range	P2 (low MoCA)	# Extreme Outliers
Tactile Detection	0.32 ± 0.06	0.23 - 0.44	0	0.27 ± 0.04	0.21 - 0.34	0.22	0
Tactile 2-Point Discrimination	0.56 ± 0.12	0.42 - 0.79	0	0.33 ± 0.07	0.19 - 0.51	0.19	0
Sensorimotor Integration	8.63 ± 2.27	4.83 - 12.11	0	5.78 ± 1.20	3.45 - 7.37	7.37	0

Supplementary Table 1. Sensorimotor performance of younger and older adults. Tactile detection (given in 1/[log10 0.1 mg]) and tactile two-point discrimination (given in 1/mm) were tested at the right index finger. Sensorimotor integration (given as precision grip accuracy in seconds) involved the thumb and the index finger. To ensure that higher values indicate better performance, tactile detection thresholds and tactile 2-point discrimination thresholds were reversed. Extreme outliers are defined as data points smaller or greater than the mean ± 3 standard deviations (SD). For the participant with the low MocA score (P2) individual values are given for the performed tasks.

condition	diff ± se	p	95% CI
qT1 M1 0-2 mm	94.06 ± 35.72	0.007	24.88, 163.22
qT1 M1 2-4 mm	42.87 ± 26.03	0.105	-8.01, 93.46
qT1 M1 4-6 mm	29.88 ± 23.60	0.217	-16.30, 75.81
qT1 M1 6-8 mm	-10.70 ± 23.33	0.660	-56.15, 34.73
qT1 M1 8-10 mm	-75.15 ± 34.96	0.029	-143.00, -8.26
qT1 S1 0-2 mm	78.28 ± 31.55	0.012	16.94, 139.73
qT1 S1 2-4 mm	42.74 ± 26.57	0.111	-8.60, 94.60
qT1 S1 4-6 mm	36. 45 ± 25.55	0.161	-13.23, 86.24
qT1 S1 6-8 mm	13.15 ± 22.71	0.578	-30.78, 57.31
qT1 S1 8-10 mm	-12.73 ± 25.08	0.618	-61.78, 36.23

Supplementary Table 2. Results of permutation Welch two sample t-tests comparing quantitative T1 (qT1) values between younger adults (n=18) and older adults (n=17). Results are given as permutation mean difference ± standard error (diff ± se) in milliseconds, permutation p-value (p) and 95% permutation confidence interval (95% CI). M1 = primary motor cortex, S1 = primary somatosensory cortex.

Effect	Conditions	Mean (ms)	SEM (ms)	Statistics				
				DFn	DFd	F	p	η_G^2
Age	Younger	1710.74	15.66	1	33	0.137	0.713	0.003
	Older	1702.41	16.12					
Brain Area	M1	1680.95	12.30	1	33	41.597	$2.585*10^{-7}$	0.086
	S1	1732.19	11.52					
Cortical Depth	Deep	1511.72	12.38	3	31	1148.357	$4.760*10^{-43}$	0.743
	Inner Middle	1649.06	11.22					
	Outer Middle	1770.39	10.10					
	Superficial	1895.13	13.94					
Distance	2.01 - 4 mm	1719.03	12.16	3	31	6.494	0.009	0.014
	4.01 - 6 mm	1712.38	11.53					
	6.01 - 8 mm	1701.47	10.89					
	8.01 - 10 mm	1693.41	12.87					
Age x Distance	Younger / 2.01 - 4 mm	1740.50	16.95	3	31	19.232	$1.447*10^{-5}$	0.040
	Younger / 4.01 - 6 mm	1728.96	16.06					
	Younger / 6.01 - 8 mm	1702.08	15.18					
	Younger / 8.01 - 10 mm	1671.42	17.94					
	Older / 2.01 - 4 mm	1697.57	17.44					
	Older / 4.01 - 6 mm	1695.80	16.53					
	Older / 6.01 - 8 mm	1700.87	15.62					
	Older / 8.01 - 10 mm	1715.39	18.46					
Age x Brain Area	Younger / M1	1697.34	17.15	1	33	2.116	0.155	0.005
	Older / M1	1682.57	17.64					
	Younger / S1	1742.14	16.06					
	Older / S1	1722.25	16.53					
Brain Area x Distance	M1 / 2.01 - 4 mm	1704.16	12.67	3	31	5.407	0.015	0.007
	M1 / 4.01 - 6 mm	1687.94	11.69					
	M1 / 6.01 - 8 mm	1671.56	11.81					
	M1 / 8.01 - 10 mm	1660.16	16.50					
	S1 / 2.01 - 4 mm	1733.91	12.96					
	S1 / 4.01 - 6 mm	1736.82	12.58					
	S1 / 6.01 - 8 mm	1731.39	11.47					
	S1 / 8.01 - 10 mm	1726.65	12.68					
Age x Brain Area x Distance	Younger / M1 / 2.01 - 4 mm	1725.63	17.66	3	31	3.714	0.024	0.005
	Younger / M1 / 4.01 - 6 mm	1702.89	16.30					
	Younger / M1 / 6.01 - 8 mm	1666.23	16.46					
	Younger / M1 / 8.01 - 10 mm	1622.62	23.00					
	Younger / S1 / 2.01 - 4 mm	1755.36	18.06					
	Younger / S1 / 4.01 - 6 mm	1755.03	17.53					
	Younger / S1 / 6.01 - 8 mm	1737.92	15.99					
	Younger / S1 / 8.01 - 10 mm	1720.23	17.68					
	Older / M1 / 2.01 - 4 mm	1682.68	18.18					
	Older / M1 / 4.01 - 6 mm	1672.99	16.77					
	Older / M1 / 6.01 - 8 mm	1676.88	16.94					
	Older / M1 / 8.01 - 10 mm	1697.71	23.66					
	Older / S1 / 2.01 - 4 mm	1712.45	18.58					
	Older / S1 / 4.01 - 6 mm	1718.60	18.04					
	Older / S1 / 6.01 - 8 mm	1724.86	16.45					
	Older / S1 / 8.01 - 10 mm	1733.07	18.19					

Supplementary Table 3. ANOVA Results for quantitative T1 (qT1) values excluding the closest distance. Results for the ANOVA on qT1 values with factors age (younger adults, older adults), cortical depth (4 depths), brain area (primary somatosensory cortex, S1; primary motor cortex, M1) and venous distance (4 distances, excluding the distance 0.01 - 2 mm). Given are mean qT1 values in milliseconds (Mean in ms), degrees of freedom of the numerator (DFn), degrees of freedom of the denominator (DFd), p-values (p), and effect sizes (nG^2). P-values ≤ 0.05 are considered as significant.

Effect	Conditions	Mean (ms)	SEM (ms)	Statistics				
				DFn	DFd	F	p	η_G^2
Age	Younger	1725.97	16.19	1	33	1.010	0.322	0.017
	Older	1702.26	17.17					
Brain Area	M1	1692.69	13.11	1	33	23.004	$3.590*10^{-5}$	0.055
	S1	1735.54	12.11					
Cortical Depth	Deep	1522.65	13.49	3	31	926.059	$1.378*10^{-37}$	0.711
	Inner Middle	1657.41	12.00					
	Outer Middle	1776.73	10.53					
	Superficial	1899.67	14.30					
Distance	0.01 - 2 mm	1745.46	13.79	4	30	25.229	$3.039*10^{-8}$	0.044
	2.01 - 4 mm	1721.05	12.38					
	4.01 - 6 mm	1713.99	11.78					
	6.01 - 8 mm	1700.61	11.20					
	8.01 - 10 mm	1689.45	12.67					
Age x Distance	Younger / 0.01 - 2 mm	1786.91	18.92	4	30	26.928	$1.286*10^{-8}$	0.047
	Younger / 2.01 - 4 mm	1740.50	16.99					
	Younger / 4.01 - 6 mm	1728.96	16.16					
	Younger / 6.01 - 8 mm	1702.08	15.36					
	Younger / 8.01 - 10 mm	1671.42	17.38					
	Older / 0.01 - 2 mm	1704.02	20.07					
	Older / 2.01 - 4 mm	1701.61	18.02					
	Older / 4.01 - 6 mm	1699.02	17.14					
	Older / 6.01 - 8 mm	1699.15	16.30					
	Older / 8.01 - 10 mm	1707.48	18.44					
Age x Brain Area	Younger / M1	1700.53	17.98	1	33	0.808	0.376	0.002
	Older / M1	1684.84	19.07					
	Younger / S1	1751.41	16.61					
	Older / S1	1719.67	17.62					
Brain Area x Distance	M1 / 0.01 - 2 mm	1739.60	16.64	4	30	7.734	$4.802*10^{-4}$	0.011
	M1 / 2.01 - 4 mm	1705.64	12.99					
	M1 / 4.01 - 6 mm	1689.10	12.01					
	M1 / 6.01 - 8 mm	1670.83	12.16					
	M1 / 8.01 - 10 mm	1658.28	16.92					
	S1 / 0.01 - 2 mm	1751.33	14.83					
	S1 / 2.01 - 4 mm	1736.47	13.12					
	S1 / 4.01 - 6 mm	1738.88	12.81					
	S1 / 6.01 - 8 mm	1730.40	11.79					
	S1 / 8.01 - 10 mm	1720.63	11.61					
Age x Brain Area x Distance	Younger / M1 / 0.01 - 2 mm	1739.60	16.64	4	30	4.868	0.007	0.007
	Younger / M1 / 2.01 - 4 mm	1705.64	12.99					
	Younger / M1 / 4.01 - 6 mm	1689.10	12.01					
	Younger / M1 / 6.01 - 8 mm	1670.83	12.16					
	Younger / M1 / 8.01 - 10 mm	1658.28	16.92					
	Younger / S1 / 0.01 - 2 mm	1751.33	14.83					
	Younger / S1 / 2.01 - 4 mm	1736.47	13.12					
	Younger / S1 / 4.01 - 6 mm	1738.88	12.81					
	Younger / S1 / 6.01 - 8 mm	1730.40	11.79					
	Younger / S1 / 8.01 - 10 mm	1720.63	11.61					
	Older / M1 / 0.01 - 2 mm	1693.90	24.22					
	Older / M1 / 2.01 - 4 mm	1685.65	18.90					
	Older / M1 / 4.01 - 6 mm	1675.31	17.47					
	Older / M1 / 6.01 - 8 mm	1675.42	17.70					
	Older / M1 / 8.01 - 10 mm	1693.93	24.62					
	Older / S1 / 0.01 - 2 mm	1714.13	21.58					
	Older / S1 / 2.01 - 4 mm	1717.57	19.09					
	Older / S1 / 4.01 - 6 mm	1722.72	18.64					
	Older / S1 / 6.01 - 8 mm	1722.88	17.16					
	Older / S1 / 8.01 - 10 mm	1721.03	16.90					

Supplementary Table 4. ANOVA Results for quantitative T1 (qT1) values without participant P2. Results for the mixed-effects ANOVA on qT1 values with factors age (n=18 younger adults, n=17 older adults), cortical depth (4 depths), brain area (primary somatosensory cortex, S1; primary motor cortex, M1) and venous distance (5 distances). Given are mean qT1 values in milliseconds (Mean in ms) and standard error of the mean (SEM), degrees of freedom of the numerator (DFn), degrees of freedom of the denominator (DFd), p-values (p), and effect sizes (n_G^2). P-values ≤ 0.05 are considered as significant.

condition	diff ± se	P	95% CI
pQSM M1 0-2 mm	-0.0076 ± 0.0021	$4*10^{-5}$	-0.0118, -0.0034
pQSM M1 2-4 mm	-0.0054 ± 0.0012	$< 10^{-5}$	-0.0078, -0.0030
pQSM M1 4-6 mm	-0.0059 ± 0.0012	$< 10^{-5}$	-0.0083, -0.0036
pQSM M1 6-8 mm	-0.0072 ± 0.0016	$< 10^{-5}$	-0.0103, -0.0041
pQSM M1 8-10 mm	-0.0073 ± 0.0019	$4*10^{-5}$	-0.0110, -0.0036
pQSM S1 0-2 mm	-0.0037 ± 0.0017	0.028	-0.0069, -0.0004
pQSM S1 2-4 mm	-0.0035 ± 0.0010	$< 10^{-5}$	-0.0053, -0.0016
pQSM S1 4-6 mm	-0.0030 ± 0.0009	0.0001	-0.0047, -0.0013
pQSM S1 6-8 mm	-0.0031 ± 0.0008	0.034	-0.0031, -0.0001
pQSM S1 8-10 mm	-0.0011 ± 0.0011	0.295	-0.0032, 0.0010

Supplementary Table 5. Results of permutation Welch two sample t-tests comparing positive QSM (pQSM) values between younger adults (n=18) and older adults (n=17). Results are given as permutation mean difference ± standard error (diff ± se) in parts per million, permutation p-value (p) and 95% permutation confidence interval (95% CI). M1 = primary motor cortex, S1 = primary somatosensory cortex.

Effect	Conditions	Mean (ppm)	SEM (ppm)	Statistics				
				DFn	DFd	F	p	η_G^2
Age	Younger	0.0125	0.00047	1	33	44.722	$1.507*10^{-7}$	0.261
	Older	0.0171	0.00050					
Brain Area	M1	0.0163	0.00041	1	33	65.728	$2.939*10^{-9}$	0.129
	S1	0.0133	0.00037					
Cortical Depth	Deep	0.0120	0.00033	3	31	143.172	$1.598*10^{-23}$	0.166
	Inner Middle	0.0148	0.00037					
	Outer Middle	0.0163	0.00042					
	Superficial	0.0161	0.00037					
Distance	0.01 - 2 mm	0.0162	0.00065	4	30	7.901	0.002	0.054
	2.01 - 4 mm	0.0140	0.00038					
	4.01 - 6 mm	0.0137	0.00030					
	6.01 - 8 mm	0.0147	0.00036					
	8.01 - 10 mm	0.0155	0.00059					
Age x Distance	Younger / 0.01 - 2 mm	0.0134	0.00089	4	30	0.629	0.500	0.005
	Younger / 2.01 - 4 mm	0.0118	0.00052					
	Younger / 4.01 - 6 mm	0.0115	0.00041					
	Younger / 6.01 - 8 mm	0.0125	0.00050					
	Younger / 8.01 - 10 mm	0.0134	0.00080					
	Older / 0.01 - 2 mm	0.0190	0.00094					
	Older / 2.01 - 4 mm	0.0162	0.00055					
	Older / 4.01 - 6 mm	0.0159	0.00043					
	Older / 6.01 - 8 mm	0.0168	0.00053					
	Older / 8.01 - 10 mm	0.0176	0.00085					
Age x Brain Area	Younger / M1	0.0130	0.00056	1	33	31.846	$3.056*10^{-6}$	0.067
	Older / M1	0.0196	0.00059					
	Younger / S1	0.0121	0.00051					
	Older / S1	0.0146	0.00054					
Brain Area x Distance	M1 / 0.01 - 2 mm	0.0177	0.00089	4	30	0.224	0.756	0.001
	M1 / 2.01 - 4 mm	0.0157	0.00044					
	M1 / 4.01 - 6 mm	0.0152	0.00033					
	M1 / 6.01 - 8 mm	0.0162	0.00051					
	M1 / 8.01 - 10 mm	0.0167	0.00074					
	S1 / 0.01 - 2 mm	0.0147	0.00082					
	S1 / 2.01 - 4 mm	0.0124	0.00039					
	S1 / 4.01 - 6 mm	0.0122	0.00036					
	S1 / 6.01 - 8 mm	0.0131	0.00038					
	S1 / 8.01 - 10 mm	0.0143	0.00055					
Age x Brain Area x Distance	Younger / M1 / 0.01 - 2 mm	0.0138	0.00123	4	30	1.942	0.161	0.009
	Younger / M1 / 2.01 - 4 mm	0.0129	0.00060					
	Younger / M1 / 4.01 - 6 mm	0.0122	0.00046					
	Younger / M1 / 6.01 - 8 mm	0.0127	0.00070					
	Younger / M1 / 8.01 - 10 mm	0.0132	0.00102					
	Younger / S1 / 0.01 - 2 mm	0.0129	0.00113					
	Younger / S1 / 2.01 - 4 mm	0.0107	0.00053					
	Younger / S1 / 4.01 - 6 mm	0.0107	0.00050					
	Younger / S1 / 6.01 - 8 mm	0.0124	0.00052					
	Younger / S1 / 8.01 - 10 mm	0.0137	0.00076					
	Older / M1 / 0.01 - 2 mm	0.0215	0.00130					
	Older / M1 / 2.01 - 4 mm	0.0184	0.00063					
	Older / M1 / 4.01 - 6 mm	0.0181	0.00049					
	Older / M1 / 6.01 - 8 mm	0.0197	0.00074					
	Older / M1 / 8.01 - 10 mm	0.0203	0.00108					
	Older / S1 / 0.01 - 2 mm	0.0165	0.00120					
	Older / S1 / 2.01 - 4 mm	0.0141	0.00056					
	Older / S1 / 4.01 - 6 mm	0.0136	0.00053					
	Older / S1 / 6.01 - 8 mm	0.0139	0.00055					
	Older / S1 / 8.01 - 10 mm	0.0148	0.00080					

Supplementary Table 6. ANOVA Results for positive QSM (pQSM) values without participant P2. Results for the ANOVA on pQSM values with factors age (n=18 younger adults, n=16 older adults), cortical depth (4 depths), brain area (primary somatosensory cortex, S1; primary motor cortex, M1) and venous distance (5 distances). Given are mean pQSM values in parts per million (Mean in ppm) and standard error of the mean (SEM), degrees of freedom of the numerator (DFn), degrees of freedom of the denominator (DFd), p-values (p), and effect sizes (n_G^2). P-values ≤ 0.05 are considered as significant.

Effect	Conditions	Mean	SEM	Statistics				
		(ppm)	(ppm)	DFn	DFd	F	p	η_G^2
Age	Younger	0.0123	0.00048	1	33	39.885	$3.824*10^{-7}$	0.337
	Older	0.0167	0.00050					
Brain Area	M1	0.0160	0.00042	1	33	78.835	$2.912*10^{-10}$	0.189
	S1	0.0130	0.00035					
Cortical Depth	Deep	0.0115	0.00028	3	31	134.910	$1.173*10^{-19}$	0.264
	Inner Middle	0.0144	0.00038					
	Outer Middle	0.0162	0.00045					
	Superficial	0.0158	0.00040					
Distance	2.01 - 4 mm	0.0140	0.00037	3	31	10.687	$4.384*10^{-4}$	0.050
	4.01 - 6 mm	0.0137	0.00029					
	6.01 - 8 mm	0.0147	0.00036					
	8.01 - 10 mm	0.0155	0.00057					
Age x Distance	Younger / 2.01 - 4 mm	0.0118	0.00051	3	31	0.052	0.911	$2.525*10^{-4}$
	Younger / 4.01 - 6 mm	0.0115	0.00040					
	Younger / 6.01 - 8 mm	0.0125	0.00050					
	Younger / 8.01 - 10 mm	0.0134	0.00079					
	Older / 2.01 - 4 mm	0.0163	0.00052					
	Older / 4.01 - 6 mm	0.0159	0.00042					
	Older / 6.01 - 8 mm	0.0169	0.00051					
	Older / 8.01 - 10 mm	0.0177	0.00082					
Age x Brain Area	Younger / M1	0.0128	0.00058	1	33	38.484	$5.306*10^{-7}$	0.102
	Older / M1	0.0192	0.00060					
	Younger / S1	0.0119	0.00049					
	Older / S1	0.0142	0.00051					
Brain Area x Distance	M1 / 2.01 - 4 mm	0.0156	0.00042	3	31	0.695	0.485	0.002
	M1 / 4.01 - 6 mm	0.0152	0.00032					
	M1 / 6.01 - 8 mm	0.0163	0.00050					
	M1 / 8.01 - 10 mm	0.0168	0.00072					
	S1 / 2.01 - 4 mm	0.0124	0.00038					
	S1 / 4.01 - 6 mm	0.0122	0.00035					
	S1 / 6.01 - 8 mm	0.0132	0.00037					
	S1 / 8.01 - 10 mm	0.0143	0.00053					
Age x Brain Area x Distance	Younger / M1 / 2.01 - 4 mm	0.0129	0.00059	3	31	8.767	0.001	0.020
	Younger / M1 / 4.01 - 6 mm	0.0122	0.00045					
	Younger / M1 / 6.01 - 8 mm	0.0127	0.00070					
	Younger / M1 / 8.01 - 10 mm	0.0132	0.00101					
	Younger / S1 / 2.01 - 4 mm	0.0107	0.00052					
	Younger / S1 / 4.01 - 6 mm	0.0107	0.00049					
	Younger / S1 / 6.01 - 8 mm	0.0124	0.00051					
	Younger / S1 / 8.01 - 10 mm	0.0137	0.00074					
	Older / M1 / 2.01 - 4 mm	0.0183	0.00061					
	Older / M1 / 4.01 - 6 mm	0.0182	0.00046					
	Older / M1 / 6.01 - 8 mm	0.0199	0.00072					
	Older / M1 / 8.01 - 10 mm	0.0205	0.00104					
	Older / S1 / 2.01 - 4 mm	0.0142	0.00054					
	Older / S1 / 4.01 - 6 mm	0.0137	0.00051					
	Older / S1 / 6.01 - 8 mm	0.0140	0.00053					
	Older / S1 / 8.01 - 10 mm	0.0148	0.00077					

Supplementary Table 7. ANOVA Results for positive QSM (pQSM) values excluding the closest distance. Results for the ANOVA on pQSM values with factors age (younger adults, older adults), cortical depth (4 depths), brain area (primary somatosensory cortex, S1; primary motor cortex, M1) and venous distance (4 distances, excluding the distance 0.01 - 2 mm). Given are mean pQSM values in parts per million (Mean in ppm), degrees of freedom of the numerator (DFn), degrees of freedom of the denominator (DFd), p-values (p), and effect sizes (nG^2). P-values ≤ 0.05 are considered as significant.

condition	younger adults	older adults
M1 0-2 mm	-0.24	-0.21
M1 2-4 mm	**-0.46**	-0.21
M1 4-6 mm	**-0.38**	0.00
M1 6-8 mm	0.05	-0.04
M1 8-10 mm	0.11	-0.14
S1 0-2 mm	-0.13	-0.24
S1 2-4 mm	**-0.31**	**-0.41**
S1 4-6 mm	-0.28	-0.19
S1 6-8 mm	**-0.37**	-0.18
S1 8-10 mm	-0.21	-0.19

Supplementary Table 8. Pearson correlation coefficients given for the correlation between quantitative T1 (qT1) and positive QSM (pQSM) values for younger adults (n=18) and older adults (n=17) for each area-by-distance condition (as shown in Supplementary Figure 3). Areas: primary motor cortex (M1), primary somatosensory cortex (S1). Venous distances in millimetres (mm): 0-2 mm, 2-4 mm, 4-6 mm, 6-8 mm, 8-10 mm. Small correlation: $r < 0.3$, moderate correlation: $r \geq 0.3$ (highlighted in bold), strong correlation: $r \geq 0.5$.

condition	diff ± se	p	95% CI
nQSM M1 0-2 mm	0.0040 ± 0.0014	0.003	0.0012, 0.0068
nQSM M1 2-4 mm	0.0030 ± 0.0008	$< 10^{-5}$	0.0015, 0.0044
nQSM M1 4-6 mm	0.0024 ± 0.0006	$< 10^{-5}$	0.0012, 0.0036
nQSM M1 6-8 mm	0.0025 ± 0.0007	0.0001	0.0011, 0.0039
nQSM M1 8-10 mm	0.0019 ± 0.0010	0.062	-0.0001, 0.0039
nQSM S1 0-2 mm	0.0032 ± 0.0012	0.005	0.0009, 0.0056
nQSM S1 2-4 mm	0.0022 ± 0.0007	0.001	0.0008, 0.0036
nQSM S1 4-6 mm	0.0021 ± 0.0006	0.0002	0.0009, 0.0032
nQSM S1 6-8 mm	0.0029 ± 0.0007	$4*10^{-5}$	0.0015, 0.0043
nQSM S1 8-10 mm	0.0031 ± 0.0009	0.0001	0.0014, 0.0049

Supplementary Table 9. Results of permutation Welch two sample t-tests comparing negative QSM (nQSM) values between younger adults (n=18) and older adults (n=17). Results are given as permutation mean difference ± standard error (diff ± se) in parts per million, permutation p-value (p) and 95% permutation confidence interval (95% CI). M1 = primary motor cortex, S1 = primary somatosensory cortex.

Effect	Conditions	Mean (ppm)	SEM (ppm)	Statistics				
				DFn	DFd	F	p	η_G^2
Age	Younger	-0.0095	0.00038	1	33	25.136	$1.772*10^{-5}$	0.177
	Older	-0.0122	0.00039					
Brain Area	M1	-0.0105	0.00031	1	33	11.737	0.002	0.016
	S1	-0.0113	0.00027					
Cortical Depth	Deep	-0.0123	0.00033	3	31	49.416	$1.068*10^{-10}$	0.162
	Inner Middle	-0.0097	0.00033					
	Outer Middle	-0.0095	0.00031					
	Superficial	-0.0121	0.00033					
Distance	0.01 - 2 mm	-0.0115	0.00054	4	30	2.048	0.138	0.011
	2.01 - 4 mm	-0.0108	0.00027					
	4.01 - 6 mm	-0.0108	0.00019					
	6.01 - 8 mm	-0.0108	0.00025					
	8.01 - 10 mm	-0.0106	0.00037					
Age x Distance	Younger / 0.01 - 2 mm	-0.0097	0.00075	4	30	1.215	0.303	0.007
	Younger / 2.01 - 4 mm	-0.0095	0.00037					
	Younger / 4.01 - 6 mm	-0.0097	0.00026					
	Younger / 6.01 - 8 mm	-0.0095	0.00035					
	Younger / 8.01 - 10 mm	-0.0093	0.00051					
	Older / 0.01 - 2 mm	-0.0133	0.00078					
	Older / 2.01 - 4 mm	-0.0120	0.00038					
	Older / 4.01 - 6 mm	-0.0119	0.00027					
	Older / 6.01 - 8 mm	-0.0121	0.00036					
	Older / 8.01 - 10 mm	-0.0118	0.00053					
Age x Brain Area	Younger / M1	-0.0091	0.00043	1	33	0.008	0.928	$1*10^{-5}$
	Older / M1	-0.0099	0.00038					
	Younger / S1	-0.0119	0.00045					
	Older / S1	-0.0126	0.00039					
Brain Area x Distance	M1 / 0.01 - 2 mm	-0.0101	0.00065	4	30	13.493	$1.296*10^{-6}$	0.037
	M1 / 2.01 - 4 mm	-0.0102	0.00028					
	M1 / 4.01 - 6 mm	-0.0105	0.00023					
	M1 / 6.01 - 8 mm	-0.0108	0.00030					
	M1 / 8.01 - 10 mm	-0.0109	0.00049					
	S1 / 0.01 - 2 mm	-0.0129	0.00056					
	S1 / 2.01 - 4 mm	-0.0113	0.00032					
	S1 / 4.01 - 6 mm	-0.0111	0.00024					
	S1 / 6.01 - 8 mm	-0.0108	0.00027					
	S1 / 8.01 - 10 mm	-0.0103	0.00037					
Age x Brain Area x Distance	Younger / M1 / 0.01 - 2 mm	-0.0081	0.00091	4	30	1.506	0.224	0.004
	Younger / M1 / 2.01 - 4 mm	-0.0088	0.00039					
	Younger / M1 / 4.01 - 6 mm	-0.0093	0.00032					
	Younger / M1 / 6.01 - 8 mm	-0.0096	0.00042					
	Younger / M1 / 8.01 - 10 mm	-0.0100	0.00068					
	Younger / S1 / 0.01 - 2 mm	-0.0112	0.00077					
	Younger / S1 / 2.01 - 4 mm	-0.0102	0.00045					
	Younger / S1 / 4.01 - 6 mm	-0.0100	0.00033					
	Younger / S1 / 6.01 - 8 mm	-0.0094	0.00038					
	Younger / S1 / 8.01 - 10 mm	-0.0087	0.00051					
	Older / M1 / 0.01 - 2 mm	-0.0121	0.00093					
	Older / M1 / 2.01 - 4 mm	-0.0117	0.00040					
	Older / M1 / 4.01 - 6 mm	-0.0117	0.00033					
	Older / M1 / 6.01 - 8 mm	-0.0120	0.00043					
	Older / M1 / 8.01 - 10 mm	-0.0118	0.00070					
	Older / S1 / 0.01 - 2 mm	-0.0145	0.00080					
	Older / S1 / 2.01 - 4 mm	-0.0124	0.00046					
	Older / S1 / 4.01 - 6 mm	-0.0121	0.00034					
	Older / S1 / 6.01 - 8 mm	-0.0123	0.00039					
	Older / S1 / 8.01 - 10 mm	-0.0118	0.00052					

Supplementary Table 10. ANOVA Results for negative QSM (nQSM) values. Results for the ANOVA on nQSM values with factors age (n=18 younger adults, n=17 older adults), cortical depth (4 depths), brain area (primary somatosensory cortex, S1; primary motor cortex, M1) and venous distance (5 distances). Given are mean nQSM values in parts per million (Mean in ppm) and standard error of the mean (SEM), degrees of freedom of the numerator (DFn), degrees of freedom of the denominator (DFd), p-values (p), and effect sizes (n_G^2). P-values ≤ 0.05 are considered as significant.

Effect	Conditions	Mean	SEM	Statistics				
		(ms)	(ms)	DFn	DFd	F	p	η_G^2
Age	Younger	-0.00949	0.00033	1	33	28.243	$7.295*10^{-6}$	0.190
	Older	-0.01198	0.00034					
Brain Area	M1	-0.01061	0.00028	1	33	1.199	0.282	0.002
	S1	-0.01086	0.00024					
Cortical Depth	Deep	-0.01296	0.00033	3	31	60.157	$9.068*10^{-12}$	0.254
	Inner Middle	-0.00981	0.00031					
	Outer Middle	-0.00900	0.00027					
	Superficial	-0.01116	0.00031					
Distance	2.01 - 4 mm	-0.01075	0.00027	3	31	0.389	0.656	0.001
	4.01 - 6 mm	-0.01079	0.00019					
	6.01 - 8 mm	-0.01082	0.00025					
	8.01 - 10 mm	-0.01058	0.00037					
Age x Distance	Younger / 2.01 - 4 mm	-0.00946	0.00037	3	31	0.328	0.697	0.001
	Younger / 4.01 - 6 mm	-0.00968	0.00026					
	Younger / 6.01 - 8 mm	-0.00948	0.00035					
	Younger / 8.01 - 10 mm	-0.00933	0.00051					
	Older / 2.01 - 4 mm	-0.01204	0.00038					
	Older / 4.01 - 6 mm	-0.01190	0.00027					
	Older / 6.01 - 8 mm	-0.01215	0.00036					
	Older / 8.01 - 10 mm	-0.01183	0.00053					
Age x Brain Area	Younger / M1	-0.00940	0.00039	1	33	0.105	0.748	$2*10^{-4}$
	Older / M1	-0.00958	0.00033					
	Younger / S1	-0.01182	0.00040					
	Older / S1	-0.01214	0.00034					
Brain Area x Distance	M1 / 2.01 - 4 mm	-0.01024	0.00028	3	31	6.589	0.003	0.014
	M1 / 4.01 - 6 mm	-0.01050	0.00023					
	M1 / 6.01 - 8 mm	-0.01080	0.00030					
	M1 / 8.01 - 10 mm	-0.01090	0.00049					
	S1 / 2.01 - 4 mm	-0.01126	0.00032					
	S1 / 4.01 - 6 mm	-0.01108	0.00024					
	S1 / 6.01 - 8 mm	-0.01083	0.00027					
	S1 / 8.01 - 10 mm	-0.01026	0.00037					
Age x Brain Area x Distance	Younger / M1 / 2.01 - 4 mm	-0.00876	0.00039	3	31	2.410	0.098	0.005
	Younger / M1 / 4.01 - 6 mm	-0.00931	0.00032					
	Younger / M1 / 6.01 - 8 mm	-0.00958	0.00042					
	Younger / M1 / 8.01 - 10 mm	-0.00996	0.00068					
	Younger / S1 / 2.01 - 4 mm	-0.01016	0.00045					
	Younger / S1 / 4.01 - 6 mm	-0.01005	0.00033					
	Younger / S1 / 6.01 - 8 mm	-0.00939	0.00038					
	Younger / S1 / 8.01 - 10 mm	-0.00871	0.00051					
	Older / M1 / 2.01 - 4 mm	-0.01172	0.00040					
	Older / M1 / 4.01 - 6 mm	-0.01168	0.00033					
	Older / M1 / 6.01 - 8 mm	-0.01203	0.00043					
	Older / M1 / 8.01 - 10 mm	-0.01185	0.00070					
	Older / S1 / 2.01 - 4 mm	-0.01236	0.00046					
	Older / S1 / 4.01 - 6 mm	-0.01212	0.00034					
	Older / S1 / 6.01 - 8 mm	-0.01227	0.00039					
	Older / S1 / 8.01 - 10 mm	-0.01182	0.00052					

Supplementary Table 11. ANOVA Results for negative QSM (nQSM) values excluding the closest distance. Results for the ANOVA on nQSM values with factors age (younger adults, older adults), cortical depth (4 depths), brain area (primary somatosensory cortex, S1; primary motor cortex, M1) and venous distance (4 distances, excluding the distance 0.01 - 2 mm). Given are mean nQSM values in parts per million (Mean in ppm), degrees of freedom of the numerator (DFn), degrees of freedom of the denominator (DFd), p-values (p), and effect sizes (nG^2). P-values ≤ 0.05 are considered as significant.

	Conditions	Mean	SEM	Statistics				
		(ppm)	(ppm)	DFn	DFd	F	p	η_G^2
Age	Younger	1819.41	14.70	1	33	1.389	0.247	0.024
	Older	1794.55	15.13					
Brain Area	M1	1692.42	12.71	4	132	289.016	$2.274*10^{-37}$	0.490
	S1	1735.63	11.73					
	SFG	1884.02	10.43					
	CMF	1877.92	10.80					
	RMF	1844.91	11.73					
Cortical Depth	Deep	1600.74	11.69	3	99	1961.016	$3.481*10^{-50}$	0.750
	Inner Middle	1771.46	11.06					
	Outer Middle	1884.28	9.64					
	Superficial	1971.44	11.48					
Distance	0.01 - 2 mm	1829.95	11.52	4	132	42.205	$8.142*10^{-12}$	0.034
	2.01 - 4 mm	1813.58	10.62					
	4.01 - 6 mm	1808.50	10.37					
	6.01 - 8 mm	1796.77	10.41					
	8.01 - 10 mm	1786.11	11.02					
Age x Distance	Younger / 0.01 - 2 mm	1857.45	16.05	4	132	24.494	$3.155*10^{-8}$	0.020
	Younger / 2.01 - 4 mm	1832.37	14.81					
	Younger / 4.01 - 6 mm	1823.99	14.46					
	Younger / 6.01 - 8 mm	1802.86	14.50					
	Younger / 8.01 - 10 mm	1780.40	15.36					
	Older / 0.01 - 2 mm	1802.45	16.52					
	Older / 2.01 - 4 mm	1794.80	15.24					
	Older / 4.01 - 6 mm	1793.02	14.88					
	Older / 6.01 - 8 mm	1790.67	14.92					
	Older / 8.01 - 10 mm	1791.82	15.81					
Age x Brain Area	Younger / M1	1700.53	17.71	4	132	1.578	0.184	0.005
	Older / M1	1751.41	16.36					
	Younger / S1	1898.83	14.54					
	Older / S1	1897.74	15.06					
	Younger / SFG	1848.56	16.35					
	Older / SFG	1684.31	18.22					
	Younger / CMF	1719.85	16.83					
	Older / CMF	1869.22	14.96					
	Younger / RMF	1858.12	15.50					
	Older / RMF	1841.28	16.83					
Brain Area x Distance	M1 / 0.01 - 2 mm	1738.28	16.18	16	528	4.045	$9.324*10^{-4}$	0.010
	M1 / 2.01 - 4 mm	1704.16	12.67					
	M1 / 4.01 - 6 mm	1687.94	11.69					
	M1 / 6.01 - 8 mm	1671.56	11.81					
	M1 / 8.01 - 10 mm	1660.16	16.50					
	S1 / 0.01 - 2 mm	1749.39	14.49					
	S1 / 2.01 - 4 mm	1733.91	12.96					
	S1 / 4.01 - 6 mm	1736.82	12.58					
	S1 / 6.01 - 8 mm	1731.39	11.47					
	S1 / 8.01 - 10 mm	1726.65	12.68					
	SFG / 0.01 - 2 mm	1903.67	12.30					
	SFG / 2.01 - 4 mm	1892.39	10.85					
	SFG / 4.01 - 6 mm	1886.74	10.09					
	SFG / 6.01 - 8 mm	1873.53	10.75					
	SFG / 8.01 - 10 mm	1863.79	12.08					
	CMF / 0.01 - 2 mm	1892.05	12.50					
	CMF / 2.01 - 4 mm	1890.35	10.20					
	CMF / 4.01 - 6 mm	1887.24	10.53					
	CMF / 6.01 - 8 mm	1871.89	11.52					
	CMF / 8.01 - 10 mm	1848.10	11.93					
	RMF / 0.01 - 2 mm	1866.37	13.74					
	RMF / 2.01 - 4 mm	1847.11	11.10					
	RMF / 4.01 - 6 mm	1843.78	11.21					
	RMF / 6.01 - 8 mm	1835.47	11.76					
	RMF / 8.01 - 10 mm	1831.86	13.04					
Age x Brain Area x Distance	Younger / M1 / 0.01 - 2 mm	1785.29	22.55	16	528	7.198	$9.514*10^{-7}$	0.017
	Younger / M1 / 2.01 - 4 mm	1725.63	17.66					
	Younger / M1 / 4.01 - 6 mm	1702.89	16.30					
	Younger / M1 / 6.01 - 8 mm	1666.23	16.46					
	Younger / M1 / 8.01 - 10 mm	1622.62	23.00					
	Younger / S1 / 0.01 - 2 mm	1788.53	20.20					
	Younger / S1 / 2.01 - 4 mm	1755.36	18.06					
	Younger / S1 / 4.01 - 6 mm	1755.03	17.53					
	Younger / S1 / 6.01 - 8 mm	1737.92	15.99					
	Younger / S1 / 8.01 - 10 mm	1720.23	17.68					
	Younger / SFG / 0.01 - 2 mm	1910.30	17.15					
	Younger / SFG / 2.01 - 4 mm	1911.40	15.13					
	Younger / SFG / 4.01 - 6 mm	1904.02	14.07					
	Younger / SFG / 6.01 - 8 mm	1889.34	14.98					
	Younger / SFG / 8.01 - 10 mm	1879.09	16.84					
	Younger / CMF / 0.01 - 2 mm	1924.67	17.42					
	Younger / CMF / 2.01 - 4 mm	1917.75	14.21					
	Younger / CMF / 4.01 - 6 mm	1911.02	14.68					
	Younger / CMF / 6.01 - 8 mm	1884.98	16.06					
	Younger / CMF / 8.01 - 10 mm	1850.27	16.63					
	Younger / RMF / 0.01 - 2 mm	1878.46	19.16					
	Younger / RMF / 2.01 - 4 mm	1851.70	15.48					
	Younger / RMF / 4.01 - 6 mm	1847.01	15.63					
	Younger / RMF / 6.01 - 8 mm	1835.84	16.39					
	Younger / RMF / 8.01 - 10 mm	1829.80	18.18					
	Older / M1 / 0.01 - 2 mm	1691.27	23.21					
	Older / M1 / 2.01 - 4 mm	1682.68	18.17					
	Older / M1 / 4.01 - 6 mm	1672.99	16.77					
	Older / M1 / 6.01 - 8 mm	1676.88	16.94					
	Older / M1 / 8.01 - 10 mm	1697.71	23.66					
	Older / S1 / 0.01 - 2 mm	1710.25	20.79					
	Older / S1 / 2.01 - 4 mm	1712.45	18.58					

Older / S1 / 4.01 - 6 mm	1718.60	18.04
Older / S1 / 6.01 - 8 mm	1724.86	16.45
Older / S1 / 8.01 - 10 mm	1733.07	18.19
Older / SFG / 0.01 - 2 mm	1897.04	17.65
Older / SFG / 2.01 - 4 mm	1873.38	15.56
Older / SFG / 4.01 - 6 mm	1869.45	14.48
Older / SFG / 6.01 - 8 mm	1857.72	15.41
Older / SFG / 8.01 - 10 mm	1848.49	17.33
Older / CMF / 0.01 - 2 mm	1859.42	17.92
Older / CMF / 2.01 - 4 mm	1862.96	14.62
Older / CMF / 4.01 - 6 mm	1863.47	15.11
Older / CMF / 6.01 - 8 mm	1858.80	16.53
Older / CMF / 8.01 - 10 mm	1845.93	17.11
Older / RMF / 0.01 - 2 mm	1854.28	19.71
Older / RMF / 2.01 - 4 mm	1842.52	15.92
Older / RMF / 4.01 - 6 mm	1840.55	16.08
Older / RMF / 6.01 - 8 mm	1835.10	16.86
Older / RMF / 8.01 - 10 mm	1833.92	18.70

Supplementary Table 12. ANOVA Results for quantitative T1 (qT1) values including the primary motor cortex (M1), primary somatosensory cortex (S1), superior frontal gyrus (SFG), caudal middle frontal cortex (CMF), and rostral middle frontal cortex (RMF). Results for the ANOVA on qT1 values with factors age (n=18 younger adults, n=17 older adults), cortical depth (4 depths), brain area (S1, M1, SFG, CMF, RMF) and venous distance (5 distances). Given are mean qT1 values in milliseconds (Mean in ms) and standard error of the mean (SEM), degrees of freedom of the numerator (DFn), degrees of freedom of the denominator (DFd), p-values (p), and effect sizes (n_G^2). P-values ≤ 0.05 are considered as significant.

	Conditions	Mean	SEM	Statistics				
		(ppm)	(ppm)	DFn	DFd	F	p	η_G^2
Age	Younger	0.01005	0.00036	1	33	51.273	$3.321*10^{-8}$	0.246
	Older	0.01370	0.00037					
Brain Area	M1	0.01631	0.00039	4	132	130.39<br>3	$1.200*10^{-32}$	0.402
	S1	0.01336	0.00036					
	SFG	0.00931	0.00026					
	CMF	0.00992	0.00039					
	RMF	0.01048	0.00028					
Cortical Depth	Deep	0.01090	0.00027	3	99	28.519	$2.896*10^{-8}$	0.035
	Inner Middle	0.01186	0.00027					
	Outer Middle	0.01225	0.00029					
	Superficial	0.01250	0.00028					
Distance	0.01 - 2 mm	0.01317	0.00045	4	132	16.256	$2.231*10^{-5}$	0.047
	2.01 - 4 mm	0.01145	0.00027					
	4.01 - 6 mm	0.01113	0.00022					
	6.01 - 8 mm	0.01155	0.00023					
	8.01 - 10 mm	0.01208	0.00032					
Age x Distance	Younger / 0.01 - 2 mm	0.01058	0.00062	4	132	5.170	0.015	0.016
	Younger / 2.01 - 4 mm	0.00964	0.00038					
	Younger / 4.01 - 6 mm	0.00944	0.00031					
	Younger / 6.01 - 8 mm	0.01000	0.00032					
	Younger / 8.01 - 10 mm	0.01062	0.00045					
	Older / 0.01 - 2 mm	0.01575	0.00064					
	Older / 2.01 - 4 mm	0.01326	0.00039					
	Older / 4.01 - 6 mm	0.01283	0.00032					
	Older / 6.01 - 8 mm	0.01310	0.00033					
	Older / 8.01 - 10 mm	0.01354	0.00046					
Age x Brain Area	Younger / M1	0.01297	0.00055	4	132	11.941	$1.689*10^{-6}$	0.058
	Older / M1	0.01207	0.00050					
	Younger / S1	0.00772	0.00037					
	Older / S1	0.00816	0.00054					
	Younger / SFG	0.00935	0.00039					
	Older / SFG	0.01966	0.00056					
	Younger / CMF	0.01465	0.00052					
	Older / CMF	0.01091	0.00038					
	Younger / RMF	0.01168	0.00056					
	Older / RMF	0.01160	0.00040					
Brain Area x Distance	M1 / 0.01 - 2 mm	0.01763	0.00087	16	528	1.944	0.093	0.014
	M1 / 2.01 - 4 mm	0.01564	0.00042					
	M1 / 4.01 - 6 mm	0.01521	0.00032					
	M1 / 6.01 - 8 mm	0.01627	0.00050					
	M1 / 8.01 - 10 mm	0.01682	0.00072					
	S1 / 0.01 - 2 mm	0.01474	0.00080					
	S1 / 2.01 - 4 mm	0.01244	0.00038					
	S1 / 4.01 - 6 mm	0.01218	0.00035					
	S1 / 6.01 - 8 mm	0.01316	0.00037					
	S1 / 8.01 - 10 mm	0.01426	0.00053					
	SFG / 0.01 - 2 mm	0.01073	0.00048					
	SFG / 2.01 - 4 mm	0.00947	0.00029					
	SFG / 4.01 - 6 mm	0.00923	0.00024					
	SFG / 6.01 - 8 mm	0.00865	0.00023					
	SFG / 8.01 - 10 mm	0.00847	0.00027					
	CMF / 0.01 - 2 mm	0.01124	0.00053					
	CMF / 2.01 - 4 mm	0.00957	0.00043					
	CMF / 4.01 - 6 mm	0.00912	0.00038					
	CMF / 6.01 - 8 mm	0.00938	0.00039					
	CMF / 8.01 - 10 mm	0.01029	0.00051					
	RMF / 0.01 - 2 mm	0.01148	0.00075					
	RMF / 2.01 - 4 mm	0.01014	0.00025					
	RMF / 4.01 - 6 mm	0.00992	0.00024					
	RMF / 6.01 - 8 mm	0.01029	0.00026					
	RMF / 8.01 - 10 mm	0.01055	0.00038					
Age x Brain Area x Distance	Younger / M1 / 0.01 - 2 mm	0.01384	0.00121	16	528	2.278	0.052	0.016
	Younger / M1 / 2.01 - 4 mm	0.01293	0.00059					
	Younger / M1 / 4.01 - 6 mm	0.01224	0.00045					
	Younger / M1 / 6.01 - 8 mm	0.01267	0.00070					
	Younger / M1 / 8.01 - 10 mm	0.01316	0.00101					
	Younger / S1 / 0.01 - 2 mm	0.01291	0.00112					
	Younger / S1 / 2.01 - 4 mm	0.01070	0.00052					
	Younger / S1 / 4.01 - 6 mm	0.01068	0.00049					
	Younger / S1 / 6.01 - 8 mm	0.01235	0.00051					
	Younger / S1 / 8.01 - 10 mm	0.01370	0.00074					
	Younger / SFG / 0.01 - 2 mm	0.00844	0.00067					
	Younger / SFG / 2.01 - 4 mm	0.00784	0.00040					
	Younger / SFG / 4.01 - 6 mm	0.00766	0.00033					
	Younger / SFG / 6.01 - 8 mm	0.00736	0.00032					
	Younger / SFG / 8.01 - 10 mm	0.00727	0.00037					
	Younger / CMF / 0.01 - 2 mm	0.00919	0.00074					
	Younger / CMF / 2.01 - 4 mm	0.00776	0.00059					
	Younger / CMF / 4.01 - 6 mm	0.00757	0.00054					
	Younger / CMF / 6.01 - 8 mm	0.00785	0.00054					
	Younger / CMF / 8.01 - 10 mm	0.00845	0.00071					
	Younger / RMF / 0.01 - 2 mm	0.00849	0.00105					
	Younger / RMF / 2.01 - 4 mm	0.00895	0.00035					
	Younger / RMF / 4.01 - 6 mm	0.00903	0.00033					
	Younger / RMF / 6.01 - 8 mm	0.00977	0.00036					
	Younger / RMF / 8.01 - 10 mm	0.01051	0.00053					
	Older / M1 / 0.01 - 2 mm	0.02142	0.00125					
	Older / M1 / 2.01 - 4 mm	0.01834	0.00061					
	Older / M1 / 4.01 - 6 mm	0.01818	0.00046					
	Older / M1 / 6.01 - 8 mm	0.01987	0.00072					
	Older / M1 / 8.01 - 10 mm	0.02047	0.00104					
	Older / S1 / 0.01 - 2 mm	0.01657	0.00115					

Older / S1 / 2.01 - 4 mm	0.01418	0.00054
Older / S1 / 4.01 - 6 mm	0.01368	0.00051
Older / S1 / 6.01 - 8 mm	0.01397	0.00053
Older / S1 / 8.01 - 10 mm	0.01483	0.00077
Older / SFG / 0.01 - 2 mm	0.01302	0.00069
Older / SFG / 2.01 - 4 mm	0.01110	0.00041
Older / SFG / 4.01 - 6 mm	0.01081	0.00034
Older / SFG / 6.01 - 8 mm	0.00993	0.00033
Older / SFG / 8.01 - 10 mm	0.00968	0.00038
Older / CMF / 0.01 - 2 mm	0.01329	0.00076
Older / CMF / 2.01 - 4 mm	0.01139	0.00061
Older / CMF / 4.01 - 6 mm	0.01068	0.00055
Older / CMF / 6.01 - 8 mm	0.01092	0.00055
Older / CMF / 8.01 - 10 mm	0.01212	0.00073
Older / RMF / 0.01 - 2 mm	0.01448	0.00108
Older / RMF / 2.01 - 4 mm	0.01132	0.00036
Older / RMF / 4.01 - 6 mm	0.01081	0.00034
Older / RMF / 6.01 - 8 mm	0.01082	0.00037
Older / RMF / 8.01 - 10 mm	0.01059	0.00055

Supplementary Table 13. ANOVA results for positive QSM (pQSM) values including the primary motor cortex (M1), primary somatosensory cortex (S1), superior frontal gyrus (SFG), caudal middle frontal cortex (CMF), and rostral middle frontal cortex (RMF). Results for the ANOVA on pQSM values with factors age (n=18 younger adults, n=17 older adults), cortical depth (4 depths), brain area (S1, M1, SFG, CMF, RMF) and venous distance (5 distances). Given are mean pQSM values in parts per million (Mean in ppm) and standard error of the mean (SEM), degrees of freedom of the numerator (DFn), degrees of freedom of the denominator (DFd), p-values (p), and effect sizes (n_G^2). P-values ≤ 0.05 are considered as significant.

	Conditions	Mean (ppm)	SEM (ppm)	Statistics				
				DFn	DFd	F	p	η_G^2
Age	Younger	-0.00928	0.00032	1	33	42.594	$2.066*10^{-7}$	0.192
	Older	-0.01232	0.00033					
Brain Area	M1	-0.01051	0.00031	4	132	8.848	$1.054*10^{-5}$	0.026
	S1	-0.01126	0.00027					
	SFG	-0.01070	0.00024					
	CMF	-0.01006	0.00037					
	RMF	-0.01147	0.00024					
Cortical Depth	Deep	-0.01004	0.00027	3	99	78.915	$1.510*10^{-13}$	0.154
	Inner Middle	-0.00954	0.00029					
	Outer Middle	-0.01061	0.00027					
	Superficial	-0.01300	0.00028					
Distance	0.01 - 2 mm	-0.01283	0.00041	4	132	60.867	$5.079*10^{-14}$	0.103
	2.01 - 4 mm	-0.01075	0.00023					
	4.01 - 6 mm	-0.01034	0.00019					
	6.01 - 8 mm	-0.01023	0.00022					
	8.01 - 10 mm	-0.00985	0.00025					
Age x Distance	Younger / 0.01 - 2 mm	-0.01084	0.00056	4	132	3.339	0.049	0.006
	Younger / 2.01 - 4 mm	-0.00940	0.00032					
	Younger / 4.01 - 6 mm	-0.00903	0.00026					
	Younger / 6.01 - 8 mm	-0.00879	0.00030					
	Younger / 8.01 - 10 mm	-0.00834	0.00036					
	Older / 0.01 - 2 mm	-0.01483	0.00058					
	Older / 2.01 - 4 mm	-0.01210	0.00032					
	Older / 4.01 - 6 mm	-0.01166	0.00027					
	Older / 6.01 - 8 mm	-0.01167	0.00031					
	Older / 8.01 - 10 mm	-0.01135	0.00037					
Age x Brain Area	Younger / M1	-0.00914	0.00043	4	132	1.922	0.122	0.006
	Older / M1	-0.00991	0.00038					
	Younger / S1	-0.00943	0.00033					
	Older / S1	-0.00825	0.00051					
	Younger / SFG	-0.00967	0.00033					
	Older / SFG	-0.01188	0.00045					
	Younger / CMF	-0.01261	0.00039					
	Older / CMF	-0.01196	0.00034					
	Younger / RMF	-0.01187	0.00052					
	Older / RMF	-0.01328	0.00034					
Brain Area x Distance	M1 / 0.01 - 2 mm	-0.01009	0.00065	16	528	10.137	$8.102*10^{-8}$	0.054
	M1 / 2.01 - 4 mm	-0.01024	0.00028					
	M1 / 4.01 - 6 mm	-0.01050	0.00023					
	M1 / 6.01 - 8 mm	-0.01080	0.00030					
	M1 / 8.01 - 10 mm	-0.01090	0.00049					
	S1 / 0.01 - 2 mm	-0.01286	0.00056					
	S1 / 2.01 - 4 mm	-0.01126	0.00032					
	S1 / 4.01 - 6 mm	-0.01108	0.00024					
	S1 / 6.01 - 8 mm	-0.01083	0.00027					
	S1 / 8.01 - 10 mm	-0.01026	0.00037					
	SFG / 0.01 - 2 mm	-0.01357	0.00050					
	SFG / 2.01 - 4 mm	-0.01064	0.00027					
	SFG / 4.01 - 6 mm	-0.01022	0.00021					
	SFG / 6.01 - 8 mm	-0.00991	0.00020					
	SFG / 8.01 - 10 mm	-0.00915	0.00025					
	CMF / 0.01 - 2 mm	-0.01299	0.00082					
	CMF / 2.01 - 4 mm	-0.01040	0.00037					
	CMF / 4.01 - 6 mm	-0.00954	0.00034					
	CMF / 6.01 - 8 mm	-0.00900	0.00035					
	CMF / 8.01 - 10 mm	-0.00838	0.00036					
	RMF / 0.01 - 2 mm	-0.01465	0.00062					
	RMF / 2.01 - 4 mm	-0.01121	0.00025					
	RMF / 4.01 - 6 mm	-0.01038	0.00023					
	RMF / 6.01 - 8 mm	-0.01059	0.00026					
	RMF / 8.01 - 10 mm	-0.01053	0.00035					
Age x Brain Area x Distance	Younger / M1 / 0.01 - 2 mm	-0.00809	0.00091	16	528	0.614	0.672	0.003
	Younger / M1 / 2.01 - 4 mm	-0.00876	0.00039					
	Younger / M1 / 4.01 - 6 mm	-0.00931	0.00032					
	Younger / M1 / 6.01 - 8 mm	-0.00958	0.00042					
	Younger / M1 / 8.01 - 10 mm	-0.00996	0.00068					
	Younger / S1 / 0.01 - 2 mm	-0.01125	0.00077					
	Younger / S1 / 2.01 - 4 mm	-0.01016	0.00045					
	Younger / S1 / 4.01 - 6 mm	-0.01005	0.00033					
	Younger / S1 / 6.01 - 8 mm	-0.00939	0.00038					
	Younger / S1 / 8.01 - 10 mm	-0.00871	0.00051					
	Younger / SFG / 0.01 - 2 mm	-0.01193	0.00070					
	Younger / SFG / 2.01 - 4 mm	-0.00953	0.00038					
	Younger / SFG / 4.01 - 6 mm	-0.00924	0.00029					
	Younger / SFG / 6.01 - 8 mm	-0.00878	0.00028					
	Younger / SFG / 8.01 - 10 mm	-0.00768	0.00036					
	Younger / CMF / 0.01 - 2 mm	-0.01088	0.00115					
	Younger / CMF / 2.01 - 4 mm	-0.00902	0.00052					
	Younger / CMF / 4.01 - 6 mm	-0.00779	0.00048					
	Younger / CMF / 6.01 - 8 mm	-0.00718	0.00049					
	Younger / CMF / 8.01 - 10 mm	-0.00640	0.00051					
	Younger / RMF / 0.01 - 2 mm	-0.01203	0.00086					
	Younger / RMF / 2.01 - 4 mm	-0.00954	0.00035					
	Younger / RMF / 4.01 - 6 mm	-0.00878	0.00033					
	Younger / RMF / 6.01 - 8 mm	-0.00903	0.00036					
	Younger / RMF / 8.01 - 10 mm	-0.00895	0.00049					
	Older / M1 / 0.01 - 2 mm	-0.01210	0.00093					
	Older / M1 / 2.01 - 4 mm	-0.01172	0.00040					
	Older / M1 / 4.01 - 6 mm	-0.01168	0.00033					
	Older / M1 / 6.01 - 8 mm	-0.01203	0.00043					
	Older / M1 / 8.01 - 10 mm	-0.01185	0.00070					
	Older / S1 / 0.01 - 2 mm	-0.01447	0.00080					

Older / S1 / 2.01 - 4 mm	-0.01236	0.00046
Older / S1 / 4.01 - 6 mm	-0.01212	0.00034
Older / S1 / 6.01 - 8 mm	-0.01227	0.00039
Older / S1 / 8.01 - 10 mm	-0.01182	0.00052
Older / SFG / 0.01 - 2 mm	-0.01520	0.00072
Older / SFG / 2.01 - 4 mm	-0.01174	0.00039
Older / SFG / 4.01 - 6 mm	-0.01120	0.00030
Older / SFG / 6.01 - 8 mm	-0.01104	0.00029
Older / SFG / 8.01 - 10 mm	-0.01063	0.00037
Older / CMF / 0.01 - 2 mm	-0.01510	0.00118
Older / CMF / 2.01 - 4 mm	-0.01179	0.00053
Older / CMF / 4.01 - 6 mm	-0.01129	0.00049
Older / CMF / 6.01 - 8 mm	-0.01083	0.00050
Older / CMF / 8.01 - 10 mm	-0.01035	0.00052
Older / RMF / 0.01 - 2 mm	-0.01726	0.00089
Older / RMF / 2.01 - 4 mm	-0.01289	0.00036
Older / RMF / 4.01 - 6 mm	-0.01198	0.00033
Older / RMF / 6.01 - 8 mm	-0.01216	0.00037
Older / RMF / 8.01 - 10 mm	-0.01211	0.00050

Supplementary Table 14. ANOVA Results for negative QSM (nQSM) values including the primary motor cortex (M1), primary somatosensory cortex (S1), superior frontal gyrus (SFG), caudal middle frontal cortex (CMF) and rostral middle frontal cortex (RMF). Results for the ANOVA on nQSM values with factors age (n=18 younger adults, n=17 older adults), cortical depth (4 depths), brain area (S1, M1, SFG, CMF, RMF) and venous distance (5 distances). Given are mean nQSM values in parts per million (Mean in ppm) and standard error of the mean (SEM), degrees of freedom of the numerator (DFn), degrees of freedom of the denominator (DFd), p-values (p), and effect sizes (n_G^2). P-values ≤ 0.05 are considered as significant.

Supplementary References

1. Mücke, M, Cuhls, H, Radbruch, L, et al. Quantitative sensorische Testung. Schmerz 28, 635–648 (2014). https://doi.org/10.1007/s00482-014-1485-4
2. Brainard DH. The Psychophysics Toolbox. *Spat Vis*. 1997;10(4):433-436.
3. Thornbury JM, Mistretta CM. Tactile sensitivity as a function of age. *J Gerontol*. 1981;36(1):34-39. doi:10.1093/geronj/36.1.34
4. Gescheider GA, Zwislocki JJ, Rasmussen A. Effects of stimulus duration on the amplitude difference limen for vibrotaction. *J Acoust Soc Am*. 1996;100(4 Pt 1):2312-2319. doi:10.1121/1.417940
5. Kalisch T, Tegenthoff M, Dinse HR. Improvement of sensorimotor functions in old age by passive sensory stimulation. *Clin Interv Aging*. 2008;3(4):673-690. doi:10.2147/cia.s3174
6. Kuehn E, Doehler J, Pleger B. The influence of vision on tactile Hebbian learning. *Sci Rep*. 2017;7(1):9069. doi:10.1038/s41598-017-09181-6
7. Pleger B, Wilimzig C, Nicolas V, et al. A complementary role of intracortical inhibition in age-related tactile degradation and its remodelling in humans. *Sci Rep*. 2016;6:27388. doi:10.1038/srep27388
8. Kalisch T, Ragert P, Schwenkreis P, Dinse HR, Tegenthoff M. Impaired tactile acuity in old age is accompanied by enlarged hand representations in somatosensory cortex. *Cereb Cortex*. 2009;19(7):1530-1538. doi:10.1093/cercor/bhn190
9. Pleger B, Dinse HR, Ragert P, Schwenkreis P, Malin JP, Tegenthoff M. Shifts in cortical representations predict human discrimination improvement. *Proc Natl Acad Sci U S A*. 2001;98(21):12255-12260. doi:10.1073/pnas.191176298
10. Vieluf S, Godde B, Reuter EM, Voelcker-Rehage C. Age-related differences in finger force control are characterized by reduced force production. *Exp Brain Res*. 2013;224(1):107-117. doi:10.1007/s00221-012-3292-4
11. Voelcker-Rehage C, Alberts JL. Age-related changes in grasping force modulation. *Exp Brain Res*. 2005;166(1):61-70. doi:10.1007/s00221-005-2342-6
12. Gryga M, Taubert M, Dukart J, et al. Bidirectional gray matter changes after complex motor skill learning. *Front Syst Neurosci*. 2012;6:37. doi:10.3389/fnsys.2012.00037
